# Pre- and In-Service Teachers’ Attitudes Toward Students With Learning Difficulties and Challenging Behavior

**DOI:** 10.3389/fpsyg.2019.00327

**Published:** 2019-02-25

**Authors:** Mireille Krischler, Ineke M. Pit-ten Cate

**Affiliations:** ^1^Department of Psychology, Giftedness Research and Education, University of Trier, Trier, Germany; ^2^Luxembourg Centre for Educational Testing, University of Luxembourg, Luxembourg City, Luxembourg

**Keywords:** teachers’ attitudes, stereotypes, judgments, challenging behavior, learning difficulties, inclusive education

## Abstract

The implementation of inclusive policies is largely dependent on teachers’ willingness to accommodate students with special educational needs (SEN) in mainstream classrooms, which is affected by their perceived competence and attitudes. This study investigated attitudes of pre- and in-service teachers toward students with two types of SEN: challenging behavior and learning difficulties. The three components of attitudes (affective, cognitive, and behavioral) were assessed using indirect and direct measures. Results revealed that teachers held negative implicit attitudes toward challenging behavior and learning difficulties, however, implicit attitudes did not vary as a function of the type of SEN. Ratings of the stereotypical dimensions warmth and competence and overall ratings of scholastic achievement were affected by professional status and type of SEN. Professional status, implicit attitudes, and stereotypical knowledge together explained 52 and 43% of the variance in teachers’ ratings of academic proficiency for students with challenging behavior and learning difficulties, respectively. Results are interpreted within the theoretical framework and implications for teacher training are discussed.

## Introduction

Following the worldwide movement toward the inclusion of students with special educational needs (SEN), education systems across the world are changing. Research has shown that teachers are crucial in enabling success for all students (Borg et al., 2011). Although research has shown that students with and without SEN benefit from inclusion in terms of improved learning outcomes, including students’ social skills, academic achievement, and personal development (Cara, 2013), educational professionals have not automatically accepted the concept of inclusion or implement inclusive practices on a daily basis (Avramidis and Norwich, 2002). Teachers’ behaviors vary as a function of the explicit identification of SEN (Hornstra et al., 2010) and may be related to their attitudes (Eagly and Chaiken, 1993). Reviews of empirical research have shown that teachers hold positive attitudes toward the ideological concept of inclusive education, whereas their attitudes toward the inclusion of individual students with specific SENs vary (de Boer et al., 2011). Teachers expressed more positive attitudes toward the inclusion of students with physical or visual impairments or learning difficulties and more negative attitudes toward the inclusion of students with intellectual disabilities or behavioral problems (de Boer et al., 2011, 2012). Similarly, Avramidis et al. (2000) showed that teachers were significantly more concerned about the inclusion of students with emotional and behavioral difficulties than about the inclusion of students with other types of SEN. Malinen et al. (2012) stressed the importance of experience in teaching students with SEN, as positive associations were found between experience and attitudes (Avramidis and Kalyva, 2007; Jordan et al., 2009). In addition, teachers with experience in inclusive education were more confident (Avramidis et al., 2000) and reported higher levels of perceived competence and expertise concerning students with SEN (Burke and Sutherland, 2004).

The current study aimed to investigate attitudes of Luxembourgish pre- and in-service teachers toward students with SEN and their influence on judgments of student scholastic achievement. In line with the priorities of the Ministry of Education (Ministère de l’Éducation nationale, de l’Enfance et de la Jeunesse, 2017), this study focused on students with challenging behavior (i.e., ADHD and related disorders) and learning difficulties (i.e., dyslexia, dyscalculia, dysphasia, or dyspraxia).

## Theoretical Background

Attitudes reflect a set of emotions, beliefs, and behaviors toward a particular attitude object (Eagly and Chaiken, 1993). The three component model of attitudes (Eagly and Chaiken, 1993) postulates that the evaluation of the attitude object includes three types of responses: (1) the affective component (i.e., feelings toward the attitude object), (2) the cognitive component (i.e., thoughts, knowledge and beliefs about the attitude object), and (3) the behavioral component (i.e., intended behavior toward the attitude object).

### Affective Component of Attitudes –Implicit Attitudes

The affective component of attitudes reflects the emotional underpinnings of an attitude, more specifically the amount of positive or negative feelings toward the attitude object (Eagly and Chaiken, 1993). In theory and research, implicit and explicit affective attitudes are considered separately. Explicit attitudes are people’s deliberate reflections of an attitude object which together shape its evaluation (Gawronski and Bodenhausen, 2006). Expressing explicit attitudes involves controlled and effortful processes, as people have to retrieve the evaluation from memory (Fazio, 1990). In contrast, implicit attitudes concern automatic evaluations that are activated when the attitude object is present (Fazio and Olson, 2003). The Motivation and Opportunity as Determinants (MODE) model (Fazio, 1990) stipulates the different pathways that determine the relationship between attitudes and behavior. More specifically, depending on available time, motivation, and resources, the relationship between attitudes and behavior may rely on automatic or controlled processes. In demanding situations, whereby people must react immediately and have limited resources or motivation to reflect on their behavior or decisions, implicit attitudes may affect perceptions (Olson and Fazio, 2009), behaviors (Sanbonmatsu and Fazio, 1990) and judgments (Fazio et al., 1986). In contrast, the relationship between attitudes and behavior mainly relies on controlled and reflective processes when people have ample time and cognitive resources available.

Although both implicit and explicit attitudes are important for our understanding of the mechanisms that can explain teachers’ judgments and behavior, research concerning teachers’ attitudes toward students with SEN has traditionally applied explicit measurement tools. This research has revealed mixed findings, and reviews have reported both positive, neutral and negative attitudes toward (the integration or inclusion) of students with SEN (Avramidis and Norwich, 2002; de Boer et al., 2011). Due to the possible sensitivity of attitudes toward students from different social groups, implicit measures are now increasingly used (e.g., Hornstra et al., 2010; Markova et al., 2016; Krischler and Pit-ten Cate, 2018; Krischler et al., 2018). Implicit measures assess the automatic evaluative responses of the individual to an attitude object and generally rely on response times, which can be considered valid indicators of implicit attitudes (Wittenbrink, 2007). Given their reliance on automatic rather than reflective processes, implicit measures can address some concerns about response bias based on strategic answers or social desirability (Fazio et al., 1986). Studies using implicit measures, revealed negative implicit attitudes toward students with SEN (Levins et al., 2005; Hornstra et al., 2010; Enea-Drapeau et al., 2012). Due to the automatic nature of emotional reactions to attitude objects, implicit attitudes may especially be relevant to study the affective component of attitudes. Although teachers are likely to invest time and cognitive resources for high stake student evaluations, at other times implicit attitudes may affect teachers’ perceptions of their students (Olson and Fazio, 2009) as well as their teaching behaviors (e.g., Hornstra et al., 2010; Glock and Kleen, 2017), as teachers often operate in highly demanding settings, in which they are required to act within strict time limits (Santavirta et al., 2007). This may be particularly true for pre-service teachers, as their lack of experience may increase work pressure as indicted by higher levels of perceived stress (Yagil, 1998).

### Cognitive Component of Attitudes –Stereotypes

The cognitive component of attitudes is defined as an individual’s mental conceptualization of the attitude object (Eagly and Chaiken, 1993) and refers to knowledge structures or stereotypes. Stereotypes reflect assimilated information (knowledge structures) about members of social groups (e.g., Fiske and Taylor, 1991), which can facilitate, but also bias, teachers’ perceptions and judgments of student achievement (Ferguson, 2003). Stereotypes can be activated by very little information, the most salient attributes or typical characteristics of the social group (e.g., Jussim et al., 1996). Stereotype knowledge generally reduces the complexity of observations and hence facilitates the speed and effectiveness of information processing. For students with SEN, typical attributes include incompetence (Rohmer and Louvet, 2009), unproductivity or dependency (Popovich et al., 2003) and warmth (Fiske et al., 2002). Stereotypes develop following systematic principles, whereby especially warmth and competence dimensions shape the people’s impressions of others (Fiske et al., 2002). Research has suggested that the valence of an interpersonal judgment is determined by initial warmth judgments, whereby competence judgments determine the extremity of the approach-avoidance tendencies resulting from that first impression (Wojciszke et al., 1993). Studies involving a general sample of adults (Krischler and Pit-ten Cate, 2018; Krischler et al., 2018) revealed that people associate differential stereotype content with students with different types of SEN (i.e., learning difficulties and challenging behavior), whereby the mixed stereotype content combinations may evoke differential responses. More specifically, the mixed stereotype content for students with learning difficulties – low in competence but neutral in warmth - may evoke paternalistic emotions (Fiske et al., 2002), whereas the mixed stereotype content for students with challenging behavior – low in both warmth and competence – could evoke resentment (Fiske et al., 2002).

### Behavioral Component – Judgments of Scholastic Achievement

Judgments of students’ scholastic achievement can be considered a core teaching behavior, affecting instructional decision-making concerning the way in which to differentiate instructional pace, support, and task difficulty (Hoge and Coladarci, 1989; Alvidrez and Weinstein, 1999). Teachers’ judgments of students’ achievements have implications for grade retention and special education entitlement decisions and hence affect students’ educational pathways (Begeny et al., 2008). Teachers’ differential expectations for different groups of students have long been discussed as a factor affecting teaching behavior (e.g., Tenenbaum and Ruck, 2007), including their judgments of scholastic performance (e.g., Rubie-Davies et al., 2006). Teachers’ expectations may stem from stereotypes and hence differ as function of certain student characteristics (Rubie-Davies et al., 2006). For example, teachers had lower expectations of the scholastic achievement of students with learning difficulties (Shifrer, 2013) or students with challenging behavior (Hafen et al., 2015). In addition, SEN labels had a negative effect on teachers’ predictions of students’ future educational success (Vlachou et al., 2014; Hafen et al., 2015).

## Research Questions and Hypotheses

To gain a better understanding of factors that affect the successful inclusion of students with SEN, we investigated how teachers perceive and judge students with SEN. The study focused on students with learning or behavioral difficulties in line with the priorities set by the Ministry of Education. Furthermore, students diagnosed with these types of SEN are most frequently included in Luxembourgish mainstream schools (Ministère de l’Éducation nationale, de l’Enfance et de la Jeunesse, 2017). Given the three-dimensional conceptualization of attitudes and the possible sensitivities involved when measuring attitudes toward persons with SEN (for a review, see Antonak and Livneh, 2000), the current study aimed to assess the affective, cognitive, and behavioral components of attitudes toward students with learning difficulties and challenging behavior using direct and indirect measures. More specifically, to assess the affective component of attitudes we used an implicit measurement tool (indirect measure), whereas for the assessment of the cognitive and behavioral components of attitudes, ratings scales were used (direct measures).

Hypothesis 1: By using an affective priming task (Fazio et al., 1986) and based on previous findings (e.g., Levins et al., 2005; Hornstra et al., 2010; Enea-Drapeau et al., 2012) we expected that teachers would hold negative implicit attitudes toward learning difficulties and challenging behavior and that attitudes would vary as a function of SEN type (e.g., de Boer et al., 2011). More specifically, as students with challenging behavior are perceived as more challenging to teach (Landrum et al., 2003), we expected that teachers would hold more negative implicit attitudes toward challenging behavior than toward learning difficulties.

Hypothesis 2: Based on the stereotype content model and previous research (e.g., Fiske et al., 2002; Krischler et al., 2018), we expected teachers to report differential stereotype content for students with challenging behavior and learning difficulties. We expected that teachers would perceive students with learning difficulties as less competent but warm, whereas perceptions of students with challenging behavior would reflect relatively low warmth and competence.

Hypothesis 3: Based on previous findings (Hornstra et al., 2010; Shifrer, 2013; Hafen et al., 2015; Pit-ten Cate and Glock, 2018) we expected that teachers’ judgments of students’ academic achievement would be below average for both students with SEN. More specifically, we expected that the judgments of Mathematical and German proficiency for both students would be significantly below average, but more pronounced for students with learning difficulties.

Hypothesis 4: In accordance with Eagly and Chaiken (1993), we expected that the affective and cognitive components of attitudes would contribute to the judgments of students’ achievement (behavioral component). More specifically, we expected that the variance in teachers’ judgments of student achievement could be explained by their implicit attitudes and stereotypical beliefs concerning students with learning difficulties and challenging behaviors.

Hypothesis 5: Based on previous findings that professional experience, and especially experience with inclusive education (e.g., Avramidis et al., 2000; Burke and Sutherland, 2004) and contact with students with SEN (Pettigrew and Tropp, 2006) had a positive influence on attitudes toward students with SEN, we finally expected differences between pre- and in-service teachers for all three components of attitudes. More specifically, we expected less negative implicit attitudes toward learning difficulties and challenging behavior, less pronounced stereotype ratings for in-service teachers compared to pre-service teachers, and hence less stereotype bias in judgments of students’ scholastic achievement.

## Materials and Methods

(Written) informed consent was obtained for all participants. Ethical guidelines were followed throughout the study. The Ethics Review Panel of the University of Luxembourg approved the studies on 13 August 2015 (ERP-15-021) and ethical guidelines were followed throughout.

### Participants

Data were collected for a Luxembourgish sample of 46 (31 female) pre-service and 35 (29 female) in-service primary school teachers enrolled in a course on inclusive education. The course addressed the educational and social inclusion of students with SEN and focused on both the cognitive processes underlying decision-making processes and knowledge, skills and strategies concerning inclusive practice. The pre-service teachers (third year bachelor students) were aged from 21 to 34 years (*M* = 23.49; *SD* = 2.89) and had less than 6 months of teaching experience. In-service teachers’ ages ranged from 24 to 49 years (*M* = 36.56; *SD* = 7.49), with a mean teaching experience of 11.62 years (*SD* = 6.82). All in-service teachers had teaching experience with students with SEN.

### Materials

#### Affective Component of Attitudes – Implicit Attitudes

An evaluative priming task (e.g., Fazio et al., 1995) was used to assess implicit attitudes toward challenging behavior and learning difficulties. This method uses response latencies and allows for analyzing the extent to which response times are affected by the prior presentation of a prime. Words were used as primes to activate types of SEN, whereby we used a string of letters “BBBBBBB” as a neutral prime, and “LEARNING DIFFICULTIES,” or “CHALLENGING BEHAVIOR” as SEN primes. Following these prime words, participants were presented with adjectives (e.g., “happy” or “evil”; adopted from Glock et al., 2013) for which they had to judge – as quickly as possible – whether it reflected a positive or negative concept. Based on the theoretical framework, participants should respond faster for trials in which the participants’ evaluations of the primed attitude object is congruent with the valence of the adjective (target) than for trials in which they are incongruent. After 10 practice trials, responses for 90 test trials were recorded, comprising of 15 trials for each of the 2 (positive vs. negative adjective) × 3 (challenging behavior vs. learning difficulties vs. neutral letter string) combinations.

#### Cognitive Component of Attitudes – Stereotypes

Participants were asked to read two different student descriptions (adapted from Lanfranchi and Jenny, 2005). One vignette described a student with challenging behavior, who’s behavior matched diagnostic criteria for attention deficit and hyperactivity disorder with associated problems in social interactions [e.g., “For a long time, (name) has been noticed for his impulsiveness, lack of concentration and pronounced restlessness”]. The other vignette described a student with learning difficulties affecting all school subjects [e.g., “For a long time, (name) has been noticed for his significant learning and performance difficulties in reading, writing, and mathematics”]. After reading each vignette, participants provided ratings of the student’s warmth and competence using the scales of Fiske et al. (2002). Warmth was assessed by ratings of “tolerance,” “warmth,” “good naturedness” and “sincerity,” and competence was measured by ratings of “competence,” “confidence,” “independence,” and “intelligence” using a six-point-Likert-scale (1 low to 6 high). The ratings were averaged to a competence and a warmth score, respectively. Cronbach alpha internal consistency coefficients for the dimensions competence and warmth were α = 0.83 and α = 0.88, respectively.

#### Behavioral Component of Attitudes – Judgments of Scholastic Achievement

Participants were asked to provide their judgments of the described student’s German language proficiency and Mathematical performance in comparison to other students his age on a six-point Likert scale ranging from 1 (not competent) to 6 (very competent).

### Procedure

Data collection took place at the beginning of the course, using individual laptops. Participants started with the evaluative priming task and were informed that they would be briefly presented with a word, which would be followed by adjectives. They were instructed to attend to the adjective and to evaluate its valence as positive or negative by pressing the “I” or “E” key on the keyboard, respectively. Trials appeared randomly with a different sequence for each participant, to control for systematic ordering effects. Participants then read a students description and completed the rating scales for stereotype dimensions and academic achievement, after which the second vignette with the same rating scales was presented. The order of the vignettes was varied systematically across participants. During the training course, participants received individual feedback concerning their implicit attitudes and general feedback (on group level) concerning the stereotype ratings and judgments of academic achievement. After the course (i.e., after continuing education credits and grades were independently assigned), participants were asked to provide their consent for using their data for research purposes.

### Data Preparation

In a first step, all trials that involved an incorrect categorization of the target and response times under 250 ms or over 1,500 ms were excluded (Hermans et al., 2002). Difference scores were computed for implicit attitudes toward “learning difficulties” and “challenging behavior,” respectively. For each participant implicit attitude scores for “challenging behavior” and “learning difficulties” were determined by first calculating the average reaction time difference for both the negative and positive target word trials following a “challenging behavior” or “learning difficulties” prime vs. the neutral prime, respectively. Then the positive attitude score was deducted from the negative attitude score to obtain difference scores, for “challenging behavior” and “learning difficulties”. Hence, positive difference scores indicate stronger positive associations (i.e., more positive implicit attitudes) as they reflect slower response times for the classification of negative words following the prime than of positive words.

The reliability of our evaluative priming task was established by calculating mean Cronbach’s alpha reliability coefficients for each adjective (positive vs. negative) × SEN prime (learning difficulties and challenging behavior) combination, whereby we treated trial blocks as items (Wentura et al., 2005). Cronbach’s alpha values ranged from 0.76 to 0.87, with α = 0.95 across all prime combinations.

## Results

### Affective Component – Implicit Attitudes

After controlling for the neutral prime, teachers implicit attitudes toward challenging behavior (*M* = −4.90, *SD* = 128.15) and learning difficulties (*M* = −26.65, *SD* = 126.67) were negative. To test for differences in attitudes as a function of SEN type and PROFESSIONAL status, a mixed ANOVA with SEN type (learning difficulties vs. challenging behavior) as a within-subject factor and PROFESSIONAL status (pre-service vs. in-service teachers) as between-subject factor was conducted. In contrast to our expectations, results revealed no significant main effects of SEN type or PROFESSIONAL status nor an interaction effect of SEN type × PROFESSIONAL status (all *p* > 0.09).

### Cognitive Component – Stereotypes

To assess the effect of SEN type and PROFESSIONAL status on ratings of stereotype dimensions, we conducted a mixed ANOVA with SEN type (learning difficulties vs. challenging behavior) and STEREOTYPE dimensions (warmth vs. competence) as a within-subject factors and PROFESSIONAL STATUS (pre-service vs. in-service teachers) as between-subject factor. Descriptive statistics for stereotype ratings are presented in Table 1. The results revealed a significant main effect of STEREOTYPE dimension, *F*(1,66) = 81.70, *p* < 0.001, $\eta_{p}^{2}$ = 0.55 and PROFESSIONAL STATUS, *F*(1,66) = 26.95, *p* < 0.001, $\eta_{p}^{2}$ = 0.29, but not for SEN, *F*(1,66) = 1.86, *p* = 0.18, $\eta_{p}^{2}$ = 0.03. The main effect of STEREOTPYE dimension reflected that participants provided overall higher warmth than competence ratings, *t*(67) = 8.30, *p <* 0.001, *d* = 1.05. The effect of PROFESSIONAL STATUS reflected higher overall (combined stereotypes) ratings for in- vs. pre-service teachers (*M* = 3.66, *SD* = 0.58) than pre-service teachers (*M* = 2.92, *SD* = 0.56), *t*(67) = 5.20, *p <* 0.001, *d* = 1.30. These effects were signified by a significant STEREOTPYE dimension × PROFESSIONAL STATUS interaction, *F*(1,66) = 6.81, *p* = 0.01, $\eta_{p}^{2}$ = 0.09. That is, in-service teachers provided higher ratings of both warmth and competence compared to pre-service teachers, *t*(66) = 5.39, *p* < 0.001, *d* = 1.38, and *t*(66) = 3.75, *p* < 0.001, *d* = 0.93, respectively.

**Table 1:** Teachers’ ratings of the stereotype dimensions warmth and competence for students with learning difficulties and challenging behavior (data in gray reflects non-significant differences between or within groups).

	Learning Difficulties		Challenging Behavior		Total	
	Mean	*SD*	Mean	*SD*	Mean	*SD*
Warmth						
Pre-service teachers	3.82	1.00	2.51	0.75	3.16	0.69
In-service teachers	4.53	0.80	3.69	0.95	4.11	0.69
Total	4.07	0.99	2.92	1.00	3.50	0.83
Competence						
Pre-service teachers	2.38	0.74	2.99	0.72	2.68	0.56
In-service teachers	2.77	0.88	3.72	0.75	3.24	0.64
Total	2.52	0.81	3.25	0.81	2.88	0.64

The analysis also revealed a significant interaction effect of SEN type × STEREOTYPE dimension, *F*(1,66) = 172.78, *p* < 0.001, $\eta_{p}^{2}$ = 0.72. As expected, the student with challenging behavior received overall higher competence ratings than the student with learning difficulties, *t*(67) = 6.13, *p <* 0.001, *d* = 0.75, but lower warmth ratings, *t*(67) = 8.58, *p <* 0.001, *d* = 1.04. Furthermore, for the student with challenging behavior the stereotype ratings of the competence dimension were significantly higher than the ratings on the warmth dimension ratings, *t*(67) = 3.52, *p* = 0.001, *d* = 0.45. In contrast, the student with learning difficulties received significantly higher warmth than competence ratings, *t*(67) = 14.43, *p <* 0.001, *d* = 1.78. No other interaction effect was significant (*p* > 0.07).

### Behavioral Component – Judgments

To assess the effect of SEN type and PROFESSIONAL status on teachers’ JUDGMENTS, we computed a mixed ANOVA with SEN type (learning difficulties vs. challenging behavior) and JUDGMENTS (math vs. German achievement ratings) as a within-subject factors and PROFESSIONAL status (pre-service vs. in-service teachers) as between-subject factor. Descriptive statistics for scholastic achievement rating are presented in Table 2. The results revealed significant main effects of SEN type, *F*(1,67) = 70.70, *p* < 0.001, $\eta_{p}^{2}$ = 0.51, and PROFESSIONAL status, *F*(1,67) = 20.93, *p* < 0.001, $\eta_{p}^{2}$ = 0.24. The main effect of SEN type indicated that participants provided significantly higher estimated combined scholastic achievement ratings for students with challenging behavior than for students with learning difficulties, *t*(67) = 8.30, *p <* 0.001, *d* = 1.01. The main effect of PROFESSIONAL status indicated that pre- and in-service teachers gave differential judgments of students’ scholastic achievement. More specifically, in-service teachers provided overall significantly higher scholastic achievement ratings than pre-service teachers, *t*(66) = 4.64, *p <* 0.001, *d* = 1.20. All other main and interaction effects were not significant (*p* > 0.15).

**Table 2:** Teachers’ judgments of students’ academic achievement for students with learning difficulties and challenging behavior (data in gray reflects non-significant differences between or within groups).

	Learning Difficulties		Challenging Behavior		Total	
	**Mean**	***SD***	**Mean**	***SD***	**Mean**	***SD***
German Language						
Pre-service teachers	2.14	0.68	3.01	0.92	2.57	0.57
In-service teachers	2.58	1.02	3.60	0.93	3.09	0.67
Total	2.30	0.84	3.21	0.96	2.75	0.65
Mathematics						
Pre-service teachers	2.07	0.62	3.07	0.92	2.57	0.59
In-service teachers	2.58	1.21	4.00	0.78	3.29	0.64
Total	2.25	0.90	3.39	0.97	2.82	0.70
Combined achievement						
Pre-service teachers	2.10	0.56	3.04	0.85	2.57	0.54
In-service teachers	2.58	0.97	3.80	0.50	3.19	0.52
Total	2.27	0.76	3.30	0.83	2.79	0.61

### Relationships Between the Different Attitude Components

To investigate how the affective and cognitive components of attitudes contributed to differences in the judgments of the students’ scholastic achievement, we conducted a hierarchical linear regression analysis. We used the affective and the cognitive attitude components as predictors and the behavioral component (average rating of scholastic achievement) as criterion. Given the differences between pre- and in-service teachers, in a first step we controlled for professional status. Results showed that professional status explained 17% of variance in judgments of student achievement for the student with challenging behavior. In the second step, we added implicit attitudes and stereotype ratings, which explained an additional 35% of variance. More specifically, the professional status and the affective and cognitive components of attitudes together could explain 52% of variance in the behavioral component of attitudes. The effect of professional status in step 1 was not significant in step 2, as differences between pre- and in-service teachers were reflected in mean differences in stereotype ratings, which in turn predicted their judgments of student scholastic achievement for the student with challenging behavior (see Table 3). Furthermore, only the competence dimension of the stereotype ratings contributed significantly to the explanation in variance in judgments of scholastic achievement.

**Table 3:** Stepwise regression analysis predicting teachers’ average scholastic achievement ratings for the student with Challenging Behavior (*N* = 66).

Predictors	Step 1		Step 2	
	β	*t*	β	*t*
Professional status	−0.41	3.55^∗^	−0.08	0.79
Implicit attitude			−0.05	0.54
Stereotype – Warmth			0.18	1.45
Stereotype – Competence			0.55	4.60^∗^

*R^2^ 0.17^∗^ for Step 1; R^2^ change = 0.35^∗^ for Step 2, ^∗^p ≤ 0.001.*

Similarly, for the student with learning difficulties, professional status alone explained 9% of variance in judgments, whereby the additional of the affective and cognitive components in the model explained an additional 34% of variance in scholastic achievement. Again, in the final model only the competence dimension of stereotype ratings contributed significantly to this prediction (see Table 4).

**Table 4:** Stepwise regression analysis predicting teachers’ average scholastic achievement rating for the student with Learning Difficulties (*N* = 68).

Predictors	Step 1		Step 2	
	β	*t*	β	*t*
Professional status	−0.30	2.58^∗^	−0.19	1.81
Implicit attitudes			0.08	0.77
Stereotype – Warmth			−0.11	0.98
Stereotype – Competence			0.65	5.79^∗∗^

*R^2^ 0.09^∗^ for Step 1; R^2^ change = 0.34^∗∗^ for Step 2, ^∗^p < 0.05, ^∗∗^p ≤ 0.001.*

## Discussion

This study investigated all three components of attitudes concerning students with different types of SEN of pre- and in-service teachers and their interrelationships. Results confirmed that teachers’ implicit attitudes toward students with SEN are generally negative. However, in contrast with our first hypothesis, implicit attitudes did not vary as a function of SEN. Although implicit attitudes are generally believed to stem from early and past experiences (Rudman, 2004), it may be that for teachers emotional reactions toward the attitude object (i.e., the student with SEN) are also determined by (stereotypical) expectations of students’ competence and estimated scholastic achievement as, especially the latter, may reflect on perceptions of professional competence and efficacy. Teacher efficacy reflects teachers’ beliefs that they can influence students’ learning, regardless of students’ learning, behavioral, or motivational difficulties (Malinen et al., 2012), and hence may be affected by the performance of their students. To this extent, our results concerning stereotypical beliefs and judgments of scholastic achievement showed that ratings of competence as well as academic proficiency in Mathematics and German were below average for both students. These results are in line with previous research indicating that the specific identification of learning difficulties or challenging behavior resulted in lower expectations of scholastic performance (Shifrer, 2013; Hafen et al., 2015) and future educational success (Vlachou et al., 2014).

In accordance with the second hypothesis, results indicated differential stereotype content for students with different types of SEN. The combined stereotype of relatively low competence and high warmth for students with learning difficulties has been associated with paternalistic emotions (e.g., pity and sympathy) and a willingness to provide help (Fiske et al., 2002), hence may incur a protective effect. In contrast, the combination of relatively neutral ratings of competence and low warmth, may reflect either a less consensual stereotype or a polarization effect, whereby different opinions, when considered on group level, cancel each other out (Fiske et al., 2002; Cuddy et al., 2008), resulting in a score in the middle of the scale. Similarly, and in line with hypothesis 3, ratings of the scholastic proficiency varied as a function of SEN, whereby teachers had lower expectation for students with learning difficulties than for students with challenging behavior. This result was not surprising given the specific difficulties of the student with learning difficulties in comparison to the student with challenging behavior.

Although the three component model of attitudes (Eagly and Chaiken, 1993) suggests associations between the affective, cognitive and behavioral components of attitudes, results of this study showed that only stereotype ratings significantly contributed to the prediction of teachers’ judgments of scholastic proficiency, therefore only partly supporting our fourth hypothesis. This finding is in line with previous research showing strong associations between stereotypical beliefs concerning specific student groups and expectations of students’ academic proficiency (Glock and Krolak-Schwerdt, 2013; Pit-ten Cate and Glock, 2018). In accordance with the stereotype content model (Fiske et al., 2002; Cuddy et al., 2008), teachers differentially rated students with challenging behavior and learning difficulties on the dimensions warmth and competence. Although warmth may be important in regards to people’s behavior toward another person, such as approach – avoidance (Fiske et al., 2002), only competence was associated with expectations concerning the scholastic achievement of the students. This is in line with previous research (Akifyeva and Alieva, 2018), showing that expectations of academic proficiency of students belonging to a minority group are associated with teachers’ perceptions of competence, not warmth. These findings confirm the complexity of the relationships between stereotypical beliefs and behavior. Future research may investigate how different dimensions of stereotypes differentially affect teachers’ interactions with their students and their expectations of the students’ academic proficiency and success. Furthermore, the lack of association between implicit attitudes and judgments of student achievement may reflect the fact that for the stereotype and the judgment measures, participants rated a fictive student with SEN, whereas for the implicit measure, we used primes of the SEN constructs.

Concerning the role of professional experience with students with SEN, our results indicate that in-service teachers generally rated students higher on both stereotype dimensions and scholastic achievement. As pre-service teachers had less than 6 months of teaching experience, they may have had little or no experience in teaching students with different types of SEN. Stereotypical beliefs and their impact on judgments of student achievement may be reduced by increased contact with members of stereotypical groups (Weber and Crocker, 1983; Pettigrew and Tropp, 2006). However, a single disconfirming exemplar could be perceived as unrepresentative of the group (Allport, 1954; Amodio and Devine, 2006), whereby stereotypical beliefs would be upheld (Klein and Kunda, 1992). Therefore, pre-service teachers may benefit from increased opportunities for contact and interaction with students with SEN during their studies, as these experiences may challenge and possibly change their beliefs (Pettigrew and Tropp, 2006). Furthermore, training programs could affect the way teachers conceptualize difference and, in particular, educational failure, which in turn may determine their responses to current changes aimed at making educational systems more inclusive (United Nations [UN], 2006). Traditional approaches to professional development have mainly reinforced the conception that inclusive education is about “special” students who will require special support which may be difficult to organize in mainstream classrooms and hence may not produce any change in teachers’ attitudes toward (the inclusion of) students with SEN (Slee, 2001). Professional development courses could instead focus on a critical discussion of the conceptualization of inclusive practice, in combination with a reflection of pedagogical issues and conditional factors. Such courses could explicitly question the processes of pathologizing “difference,” while constructively challenge traditional educational thinking and practices (Avramidis, 2006). Previous research has shown that introducing teachers to social psychological theories, especially clarifying the processes and consequences of stereotyping, reduced bias in their decision making (Krolak-Schwerdt et al., 2018). Similarly, although attitudes are usually seen as relatively stable constructs, studies have shown that even small interventions can have positive effects (Shade and Stewart, 2001; Campbell et al., 2003; Sharma et al., 2008). In addition, teacher training programs could incorporate field work, allowing pre-service teachers to interact with students with SEN (Campbell et al., 2003), which in turn may result in reduced bias toward specific groups of students.

### Limitations and Directions for Future Research

When interpreting the results, some limitations of our study should be considered. In our study, we only presented vignettes describing male students. Stereotypical expectations concerning male students with different types of SEN may be more prominent than for female students, as male students are more often identified as having SEN and are often judged less favorably (Maniadaki et al., 2003; Holder and Kessels, 2017). Future research could, however, also consider female students, especially as a recent study has indicated teachers’ expectations for minority group students varied by gender (Kleen and Glock, 2018). Second, we only varied the two types of SEN (i.e., learning difficulties and challenging behavior) that may be the most common in mainstream classrooms, but do not do fully represent the heterogeneity of student populations. Future research could investigate attitudes toward other groups of students with SEN or the combined effects of different student’ variables (e.g., immigrant background, socio-economic status, gender, and SEN) on teachers’ expectations of academic achievement. Third, the in-service teachers in our study took part in an optional course on inclusive practice, hence their attitudes may have been more positive than those of some of their colleagues. Studies have shown that the willingness to take part in professional development courses results in less resistance to (Dickens-Smith, 1995; Leyser and Tappendorf, 2001) and less stress associated with (Forlin, 2001) the implementation of inclusive practice. The differences between pre- and in-service teachers may partly reflect this notion. As in-service teachers voluntarily signed up for the course, whilst pre-service teachers took their course as a mandatory requirement of their training program, differences in their stereotypical beliefs and expectations may be indicative of differences in attitudes toward students with SEN resulting from experience, although they may also be derived from a selection bias. Future research could investigate the role of experience vs. special interest or affinity with students with SEN on attitudes, for example by recruiting experienced teachers opting for other courses or by including pre-service teachers after completing their practice semester in inclusive settings.

Finally, in our study, teachers’ stereotypical beliefs explained variance in their ratings the mathematical and German achievement of both students. However, their judgments did not rely on actual student performance but on descriptions of fictive students. Although vignettes allow for experimental designs, which enable studying attitudes under relatively controlled conditions, teachers may react differently in response to real life situations (Lucas et al., 2009). Future research could present participants with curriculum-based schoolwork tasks in order to investigate whether implicit attitudes and stereotypical beliefs indeed influence grading (see for example Bonefeld and Dickhäuser, 2018).

## Conclusion

The study showed that teachers have differential attitudes toward students with challenging behavior and learning difficulties. Different components (i.e., affective, cognitive, and behavioral) of attitudes were distinguished, whereby in-service teachers, actively involved in teaching students with SEN, provided more positive ratings of stereotype dimensions and expectations of academic proficiency than pre-service teachers. The study emphasizes the importance of the assessment of the three components of attitudes to gain a better understanding of their different contributions to teachers’ judgments. Strong associations existed between stereotypical beliefs and judgments of scholastic achievement. These findings indicate that attitudes affect key aspects of the teaching profession and may change with experience. Especially (positive) interactions with students with SEN in mainstream classes may be pivotal in the development of more positive attitudes. It is therefore important that policy-makers and program directors enable such progression by developing pre- and in-service training programs that enhance teachers’ knowledge and skills in teaching all students and facilitate the development of positive attitudes and beliefs (Borg et al., 2011). The association between stereotypical beliefs and judgments of academic proficiency illustrates why attitudes are considered important for the successful inclusion of students with SEN in mainstream classrooms (Avramidis et al., 2000; Borg et al., 2011). In addition, a better understanding of the relationships between teachers’ attitudes and judgments of student achievement may contribute to the development of teacher training programs aimed at reducing educational disparities.

## Author Contributions

All authors are responsible for the research reported here, participated in the concept and design, analysis and interpretation of data, and drafting or revising of the manuscript, approved the manuscript as submitted, and agreed to be accountable for all aspects of the work.

## Conflict of Interest Statement

The authors declare that the research was conducted in the absence of any commercial or financial relationships that could be construed as a potential conflict of interest.
